# Congenital heart disease research landscape in the Arab world: a 25-year bibliometric review

**DOI:** 10.3389/fcvm.2023.1332291

**Published:** 2024-01-11

**Authors:** Fouad Bitar, Mariam Arabi, Ziad Bulbul, Georges Nemer, Yehya Jassar, Fadi F. Bitar, Zahi Abdul Sater

**Affiliations:** ^1^ Department of Pediatrics and Adolescent Medicine, American University of Beirut-Medical Center, Beirut, Lebanon; ^2^ Department of Pediatrics and Adolescent Medicine, Children’s Heart Center, American University of Beirut-Medical Center, Beirut, Lebanon; ^3^ Genomics and Precision Medicine (GPM), College of Health and Life Sciences at Hamad Bin Khalifa University, Doha, Qatar; ^4^ Department of Public Health, College of Public Health, Phoenicia University, Mazraat El Daoudiyeh, Lebanon; ^5^ Global Health Institute, American University of Beirut, Beirut, Lebanon

**Keywords:** research, Arab countries, limited resource countries, developing and developed countries, congenital heart disease, pediatric cardiology, children

## Abstract

**Background:**

While research on congenital heart disease has been extensively conducted worldwide, comprehensive studies from developing countries and the Arab world remain scarce.

**Aim:**

This study aims to perform a bibliometric review of research on congenital heart disease in the Arab world from 1997 to 2022.

**Methods:**

We analyzed data from the Web of Science, encompassing various aspects such as topics, countries, research output, citations, authors, collaborations, and affiliations. This comprehensive science mapping analysis was done using the *R* statistical software's Bibliometrix Package.

**Results:**

The research output from Arab countries over the 25 years showed an average annual growth rate of 11.5%. However, Arab countries exhibited lower research productivity than the United States and Europe, with a 24-fold difference. There was substantial variation in research output among 22 Arab countries, with five countries contributing to 78% of the total publications. Most of the published research was clinical, with limited innovative contributions and a preference for regional journals. High-income Arab countries displayed higher research productivity and citation rates than their low-income developing counterparts. Despite being categorized as upper-middle-income, post-conflict countries exhibited low research productivity. About one-quarter of the published articles (26%) resulted from collaborative efforts among multiple countries, with the United States being the most frequent collaborator. Enhanced research productivity and impact output were strongly associated with increased international cooperation.

**Conclusion:**

Research productivity in the Arab region closely correlates with a country's GDP. Success hinges on governmental support, funding, international collaboration, and a clear research vision. These findings offer valuable insights for policymakers, educational institutions, and governments to strengthen research programs and nurture a research culture.

## 1 Introduction

Congenital heart disease (CHD) is a prevalent anomaly affecting 1% of live births, resulting in approximately 1.5 million cases annually (1, 2). It is a primary cause of infant mortality, especially in economically disadvantaged regions, such as Low- and Middle-Income Countries (LMICs) within the Arab World (2–6). The Arab world, a diverse region comprising 22 countries, faces unique healthcare challenges in pediatric cardiology. These challenges encompass a high incidence of congenital heart disease, further compounded by elevated rates of consanguinity (6) and other cardiac disorders influenced by lifestyle factors and healthcare disparities (7, 8). Addressing these complex issues requires a solid knowledge base shaped by robust research and academic discourse. Although there is an abundance of global research on this topic, there is a notable lack of comprehensive studies that focus on the contributions and challenges in the Arab world. As healthcare systems across the Arab region rapidly evolve, assessing scientific output in various medical fields, including pediatric cardiology, assumes pivotal significance. This evaluation will provide invaluable guidance for academic initiatives and the assessment of their regional impact.

Bibliometric analysis offers a quantitative approach to evaluating research output, capturing key metrics such as publication trends, authorship patterns, and citation impact. By conducting a bibliometric analysis of research on congenital heart disease in the Arab world, we can assemble insights invaluable for researchers, policymakers, and healthcare professionals to make informed decisions and allocate resources effectively. A study focusing on improving pediatric and congenital cardiac disease care has underscored the limited progress in developing countries (9). This underscores the critical importance of conducting a specialized bibliometric analysis focused on pediatric cardiology research in developing nations and the Arab world to address this research gap.

This article aims to conduct a 25-year bibliometric review of research on congenital heart diseases in the Arab world, examining key metrics such as publication output, citation impact, collaboration networks, and thematic focuses. By synthesizing this data, the study provides a comprehensive overview of the region's contributions to the global scientific community in pediatric cardiology. This thorough analysis will not only highlight the strengths and obstacles within the scientific contributions of the Arab world but will also lay the foundation for future research collaborations, policy implications, and academic directions in this critical area of healthcare.

## 2 Materials and methods

Our study aimed to shed light on the state of research in congenital heart disease among the Arab countries, offering insights into regional and global perspectives.

### 2.1 Source of data

A bibliometric review was conducted to understand the congenital heart disease research landscape in the Arab world. Bibliometrics is a statistical analysis and quantitative tool to evaluate scientific publications' growth, impact, and trends. The Web of Science (WoS) was the source of the bibliographic data, as it contains the most comprehensive information about scientific research and is the most used in bibliometric analysis (9).

For this study, we included the following Arab countries: (Algeria OR Bahrain OR Comoros OR Djibouti OR Egypt OR Iraq OR Jordan OR Kuwait OR Lebanon OR Libya OR Mauritania OR Morocco OR Oman OR Palestinian territories OR Qatar OR Saudi Arabia OR Somalia OR Sudan OR Syria OR Tunisia OR United Arab Emirates OR Yemen OR Syrian Arab Republic).

### 2.2 Search strategy

A comprehensive search of all publications in Pediatric Cardiology was carried out from Jan 1, 1997 until December 31, 2022. A search strategy was developed by compiling an extensive list of congenital heart disease and Pediatric cardiology-related keywords identified from previous studies, reviews, and meta-analyses. Boolean operators (AND, OR, NOT) were used to conduct the search. The search strategy did not include any restrictions on the demographics of participants included in the articles (age, sex, etc.).

### 2.3 Inclusion criteria

We included articles in our selection if they had at least one author affiliated with an institution located in a country that is part of the Arab world. The dataset not only encompasses Arab countries but also includes non-Arab countries. This inclusion accounts for instances where articles authored by individuals from Arab countries also have co-authors from non-Arab countries The period of the index date was January 1, 1997, to December 31, 2022. The document type included was articles and early access documents, with the language restricted to English. All other documents, including review articles, editorials, books, and letters, were excluded.

### 2.4 Data management and selection process

The final list of references was extracted and then analyzed. The analysis was completed using the Bibliometrix Package, an *R* statistical software package for comprehensive science mapping analysis (10). The raw data exported from *R* was transformed into graphics and tabular format using the Flourish software to generate the following information: (a) Annual scientific production and article citation; (b) journals in which researchers publish; (c) country-specific production; (d) author's countries and affiliations; (e) collaboration patterns; and (f) author's keywords and title co-occurrence.

To assess research productivity in the Arab world relative to the USA, Canada, and specific European countries, we analyzed the total number of publications generated during the same timeframe. The findings were normalized to account for differences in population size.

### 2.5 Ethical approval

This study is exempt from the American University of Beirut Institutional Review Board (IRB) since the researchers used publicly available information and did not involve any interactions with human participants.

## 3. Results

### 3.1 Research productivity

Publications output for the 25 years covering 1997–2022 from the Arab countries was 2,666 articles, with an average of 5.7 articles per million population. The average number of publications for Canada, the USA, the U.K., and France were 184, 131,119, and 94 articles per million population during the same period, respectively. On average, our study found that Arab countries exhibited a research productivity per capita that was 24 times lower than that of the United States and Europe, with a range spanning from 15 to 32 times less. The average number of publications per million populations for Pakistan, India, and Nigeria, classified as non-Western and non-Arab lower-middle-income countries, was 2.4, 2.4, and 0.78 articles, respectively. South Africa, categorized as an upper-middle-income country, exhibited a publication rate of 7.7 articles per million. When reporting productivity based on the number of medical schools per region or country, the publication output from Arab countries averaged 2,666 articles across 150 medical schools, resulting in an average of 17.7 articles per medical school. In comparison, the average number of publications for Canada, the USA, the U.K., and France during the same period was 411, 282, 180, and 170 articles per medical school, respectively. On average, our study revealed that Arab countries exhibited research productivity per medical school that was 15 times lower than that of the United States and Europe, with a range spanning from 9.6 to 23 times less.

**Figure 1:**
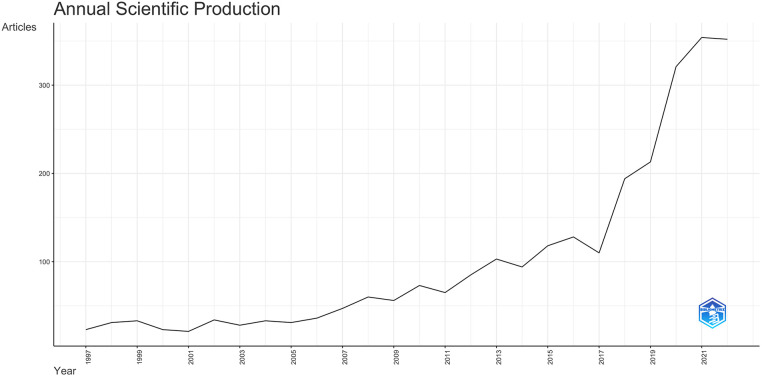
The annual scientific production relating to pediatric cardiology for the Arab countries.

There was a 4.1-fold increase in publication productivity in the Arab world over the last ten years and a 15.3-fold increase in the 25 years. The average annual growth rate was 11.5% (Figure 1).

### 3.2 Distribution of publication within the Arab countries

78% of the Arab countries' publication output was produced by five countries (Saudi Arabia, Egypt, Morocco, Lebanon, and Tunisia). Saudi Arabia had the highest percentage contribution of published articles, comprising 23.5% (*n* = 626 articles), followed by Egypt at 21.5% (*n* = 573 articles).

Qatar had the highest average of published articles per million population (24.8), followed by Saudi Arabia (17.2) and Lebanon (16.2). Gulf Cooperation Council countries, such as Kuwait and Oman, had a publication productivity of an average of 14 articles and 15 articles per million population (pmp), respectively. The North African Arab countries Egypt, Morocco, Tunisia, and Algeria, lower-middle income countries, had an average productivity of 5.2, 2.7, 7, and 0.2 articles pmp, respectively. Jordan, a lower-middle-income country in the Middle East, had an average productivity of 4.7 pmp. The low-income countries such as Yemen, Syria, Somalia, and Sudan averaged 0.5 articles per million. In comparison, post-conflict countries like Iraq and Libya had 0.9 articles pmp and 0.3 articles pmp, respectively, although they rank as upper-middle-income countries.

### 3.3 Citations

**Figure 2:**
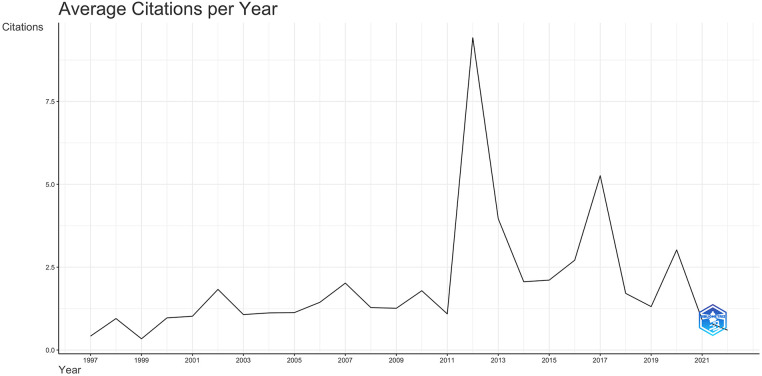
The average citation per year relating to pediatric cardiology for the Arab countries.

The mean total citation per year per article in 1997 was 0.42, 9.4 in 2012, 5.2 in 2017, and 3 in 2020. The average citation per article per year was two (Figure 2).

**Figure 3:**
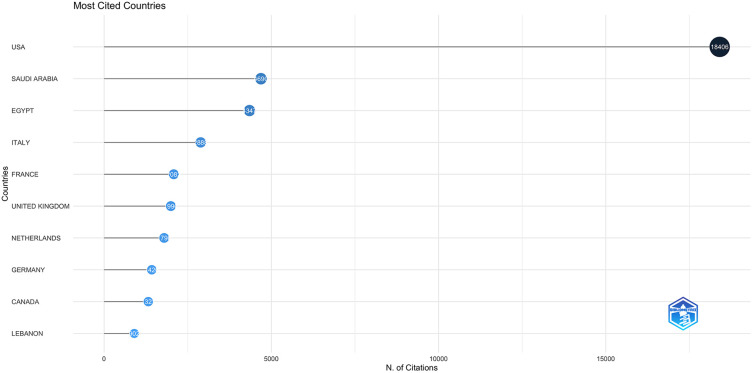
The citation frequency for countries included in the dataset. The citation frequency refers to the number of times an article was cited while the author of this article is affiliated with the noted country.

The most cited Arab countries were Saudi Arabia, Egypt, and Lebanon, with total citations of 4,690, 4,347, and 902, respectively. Lebanon had the highest citation per article at 10.1, followed by Egypt (7.6) and Saudi Arabia (7.5) (Figure 3). The low-income countries Somalia, Syria, and Yemen had an average citation of 0.5,1 and 0.8 per article. Iraq had a citation per article of 2.2. In the articles that involved multiple country publications, the most cited country was the USA, with a total citation of 18,406 and an average citation per article of 76.7, followed by Italy (22.1), France (28), Germany (24.6), the UK (20.6), and Canada (80.2).

### 3.4 Analysis of source

**Figure 4:**
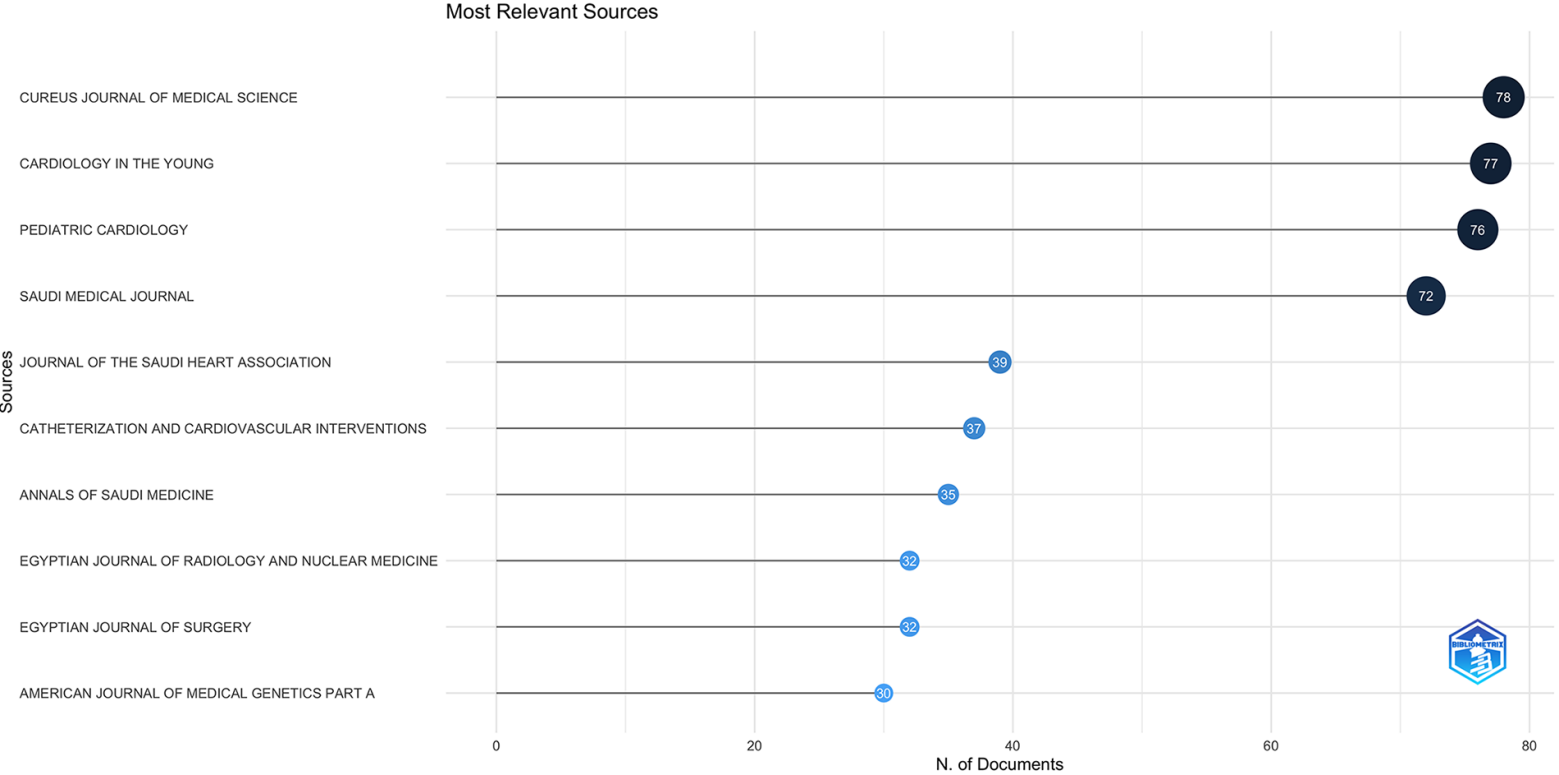
The most frequent journals involved in the publications of the articles.

Most published articles were reported in journals with relatively low or average impact factors ranging from 0.1 to 1.7. The ten most common journals in which the articles were published included three journals published in Saudi Arabia and two published in Egypt. Most published articles were clustered in journals in Quartile 3 (*Q*3), with very few published in Quartile 1 and *Q*2 (Figure 4). Journals Quartile categorizes academic journals based on their impact factors or other scholarly influence and importance metrics. The quartile classification divides journals into four groups, with Quartile 1 (*Q*1) being the highest and Quartile 4 (*Q*4) being the lowest.

### 3.5 Analysis of international collaborations

**Figure 5:**
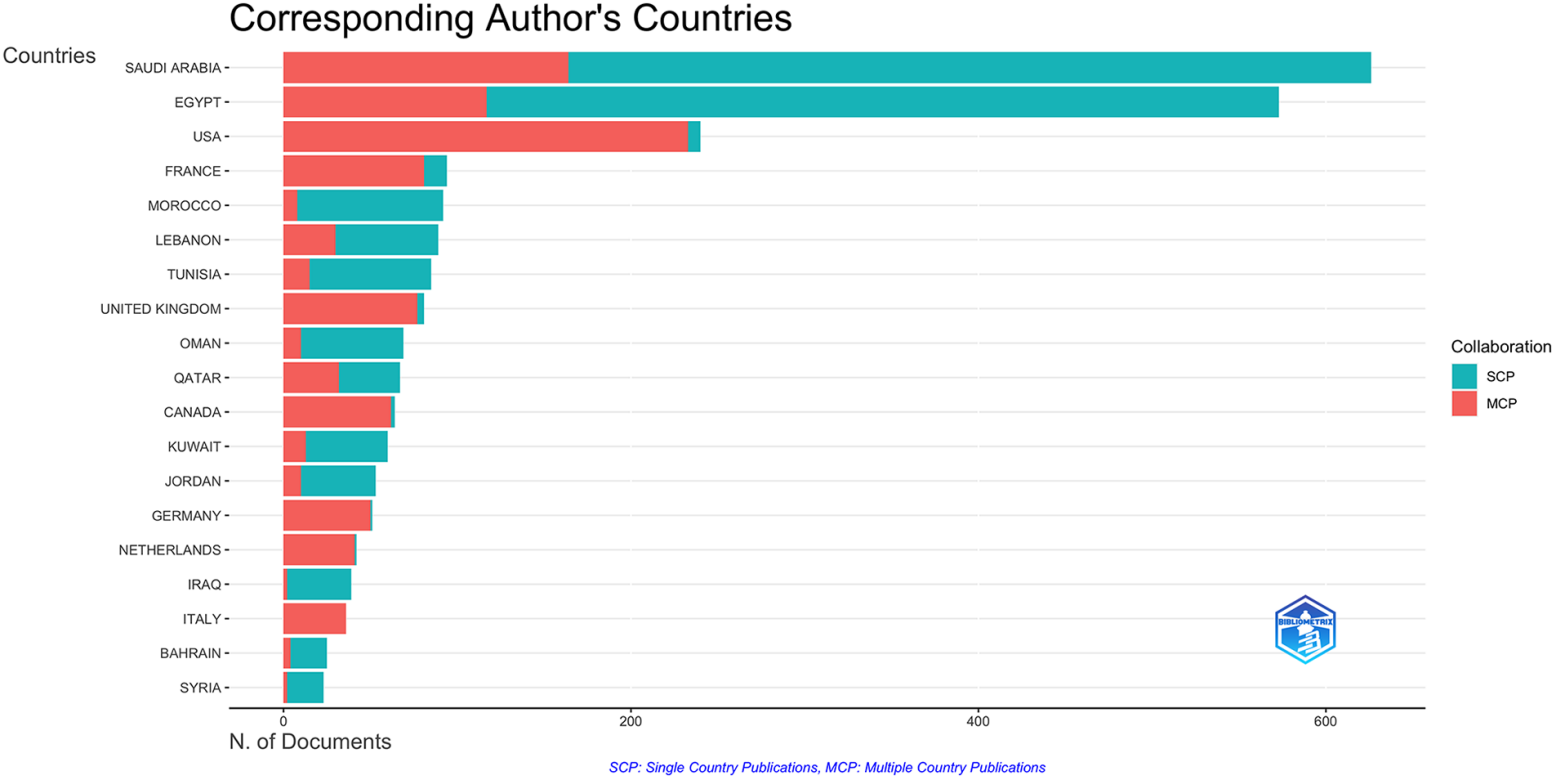
The corresponding author's countries and the relative frequency of single country publications vs. multiple countries publications.

**Figure 6:**
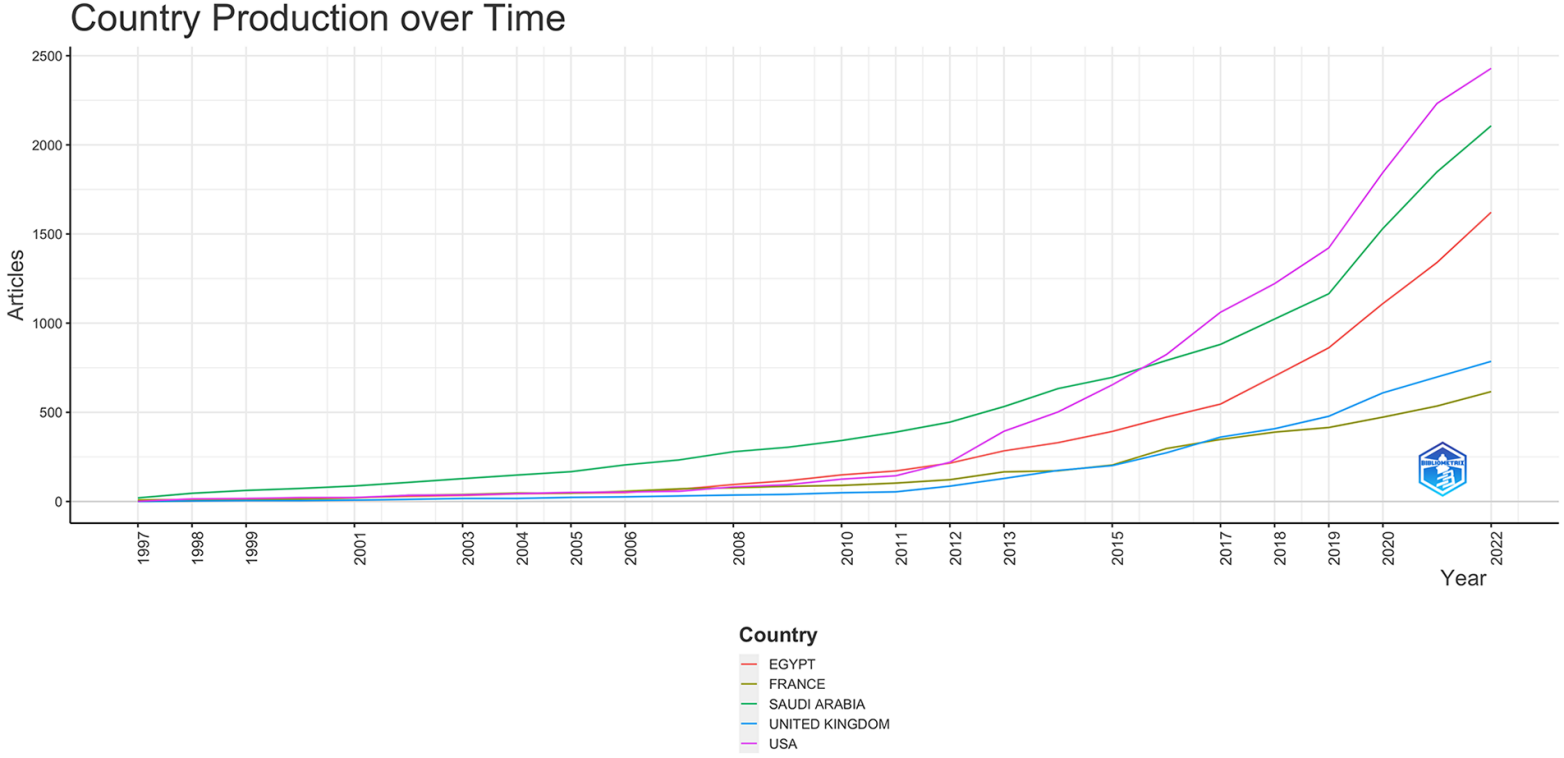
The country's publication output over time.

Of the 2,666 published articles, an average of 26% involved multiple countries and international co-authorship. Articles published from the UAE had 67% international collaboration. 48% of articles published from Qatar had multiple country production, while Lebanon had 34%, Saudi Arabia had 26%, and Egypt had 20%. Collaborations were most frequently encountered, with the USA at 35%, followed by France (14%), the UK (12%), Canada (9%), and Germany (7%) (Figures 5, 6). As the frequency of the international collaborations increased, the research productivity increased (Figure 6).

### 3.6 Analysis of affiliations, universities, and funding

**Figure 7:**
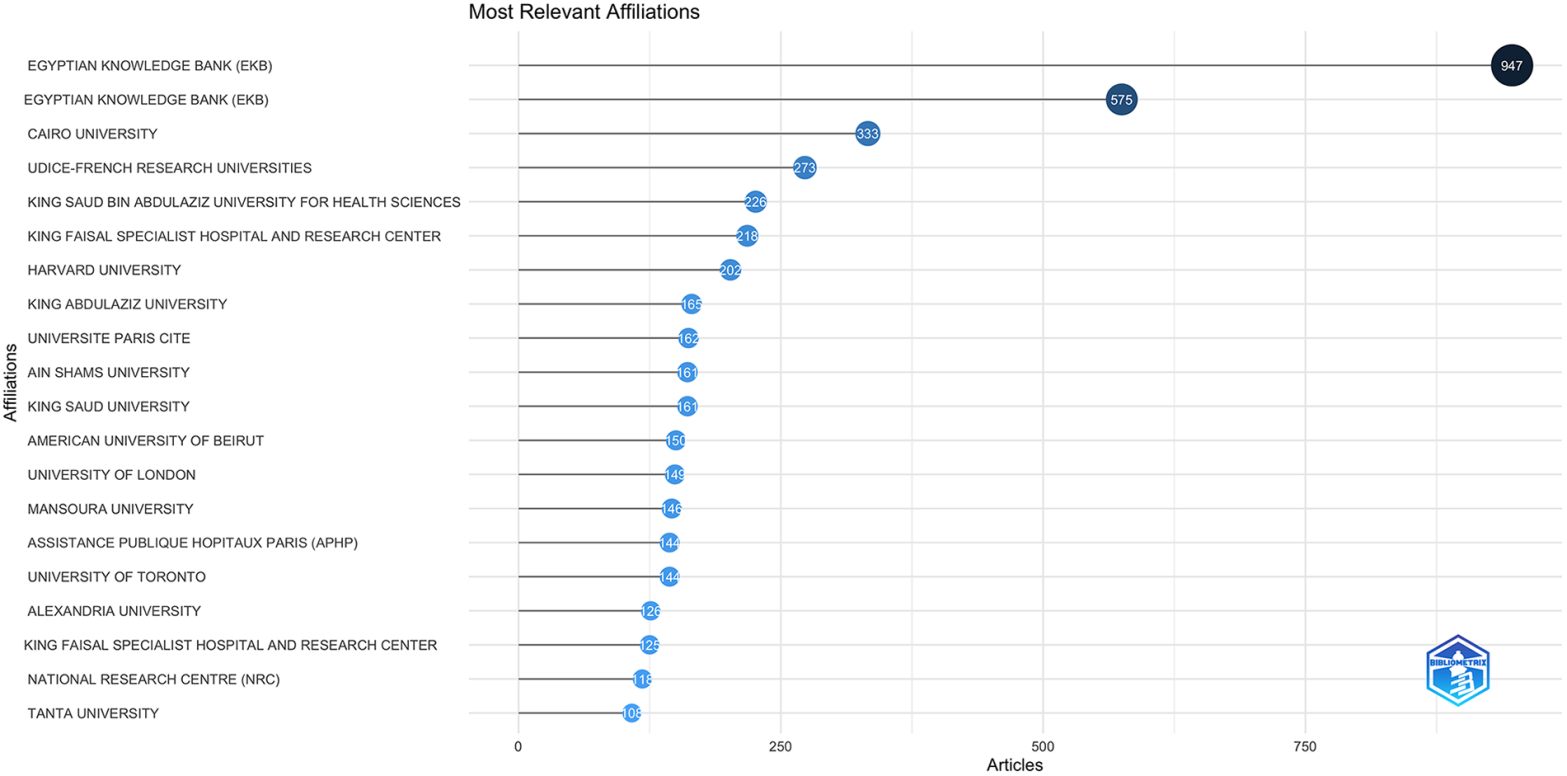
The frequency of the affiliative and universities involved in the publications.

Among the most relevant and frequent affiliative and universities involved in the publications, seven were from Egypt, with the most productive affiliate being the Egyptian Knowledge Bank (EKB), four from Saudi Arabia, one from Lebanon, two from the USA, four from France, and one each from Canada and the UK (Figure 7). We identified several good-caliber research programs that were well-funded and excelled in interdisciplinary research led by renowned experts. These programs demonstrated excellence in education, research, and global partnerships. These regional pockets of good-caliber research programs had relatively higher publication numbers representing universities from Egypt, Saudi Arabia, and Lebanon, with the universities from the high-income countries having articles with higher impact and citations.

**Figure 8:**
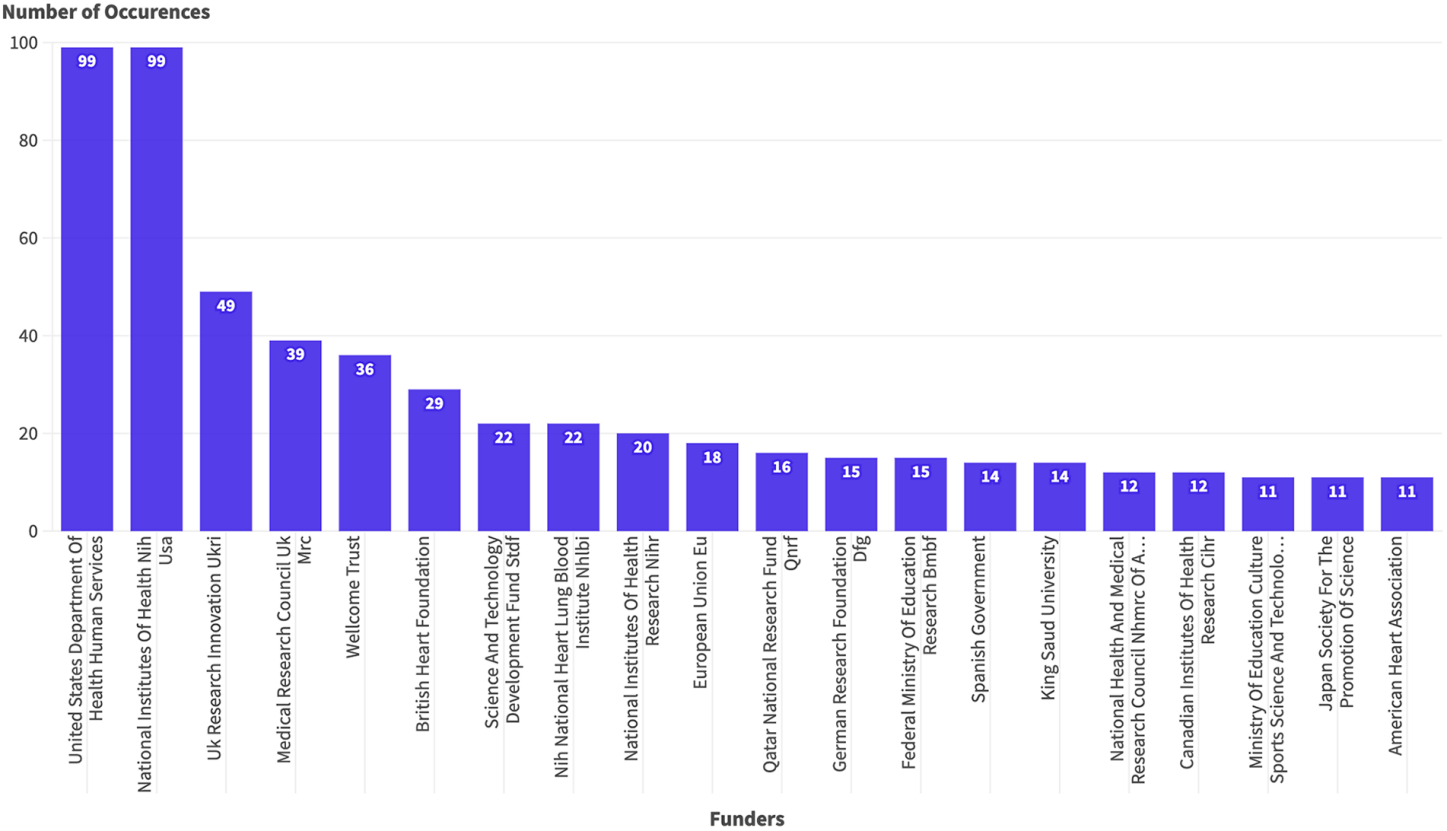
The frequency of the funders for the research in the Arab countries.

564 out of the 2,666 published articles were funded (22%), and most of the funding came from the USA and Europe, with less than 10% of the funds coming from regional sources such as the Qatar National Research Fund and regional universities (Figure 8).

### 3.7 Author's keyword and title co-occurrence

**Figure 9:**
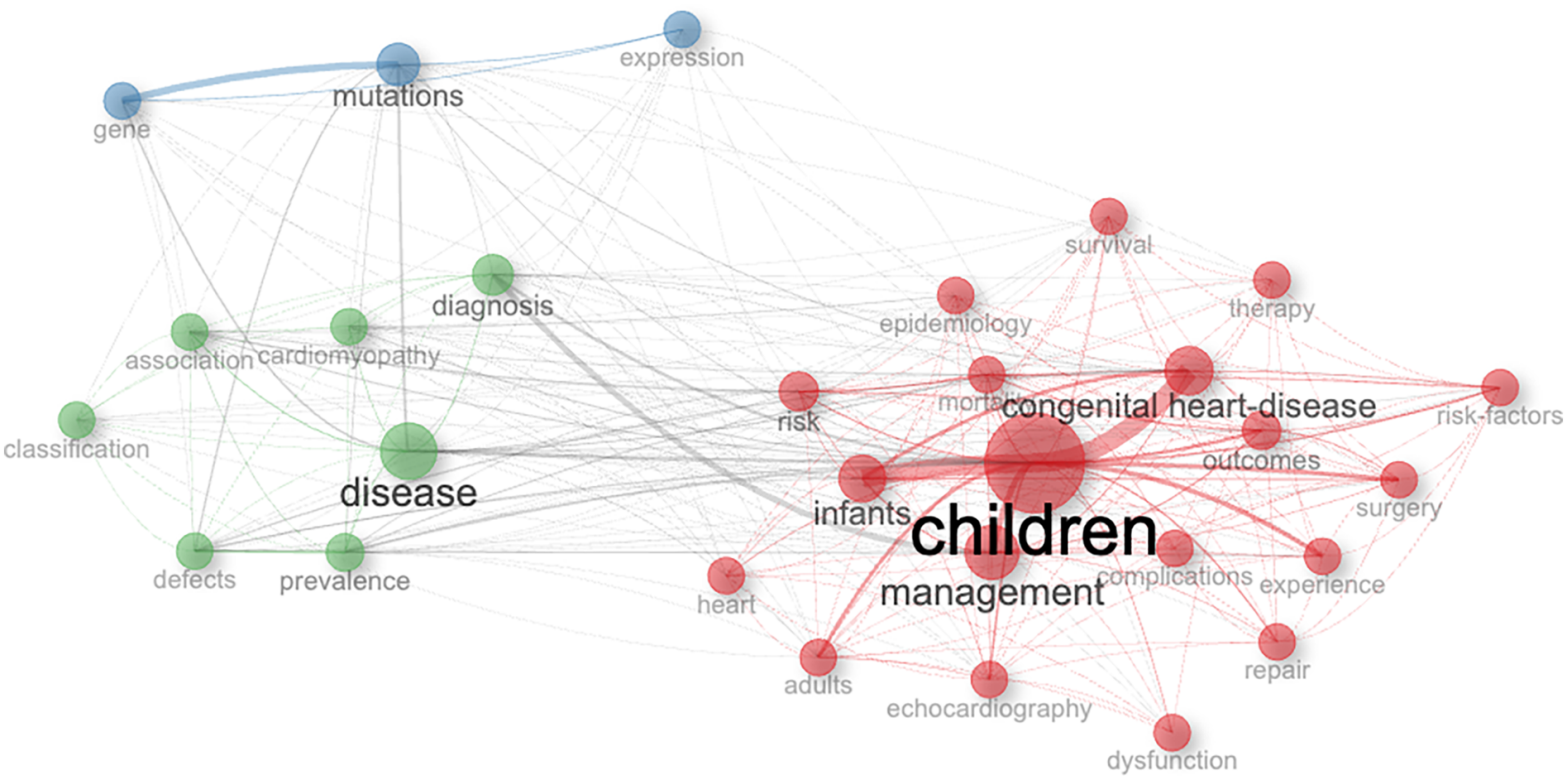
Co-occurrence by authors’ keywords on pediatric and congenital heart disease research from 1997 to 2022. The circle diameter represents the occurrence of the keyword. The edge size is proportional to item co-occurrence. Co-occurring keywords are grouped under different colors.

Research has been predominantly clinical research. The author's keywords and title co-occurrence were analyzed to characterize the research further. A world cloud was used to summarize keywords and their occurrences. The most used keywords were the management of children with congenital heart disease. Other topics included diagnosis, surgery, and outcomes of CHD. Epidemiology and prevalence of CHD and the study of cardiomyopathy were less frequently utilized. Heart disease's genetic and molecular basis were the least addressed topics (Figure 9) (9). Notably, out of the 2,666 published articles, 109 (4.1%) addressed adults with CHD. When assessing research productivity related to adult cardiology in the Arab world during the same period, 44,338 articles were published, marking a 16.6-fold increase compared to research on congenital heart disease.

### 3.8 Missing data

The missing data for the various categories was minimal. The assessment for obtaining the data without missing data revealed an excellent status for document type, journal, citation, and author, good for affiliations and corresponding author, and acceptable for keywords, abstract, and DOI.

## 4 Discussion

Research in pediatric cardiology is the cornerstone for state-of-the-art pediatric cardiology programs, enhancing our understanding of diseases and improving treatment modalities. Pediatric cardiology research has been extensive globally, but comprehensive studies from developing countries are scarce, particularly in the Arab world. Adapting educational and research initiatives in developing countries reduces mortality and complications following congenital heart surgery (11).

This study evaluated research productivity, quality, and patterns across Arab countries in pediatric and congenital heart disease. We conducted a comparative analysis between the various Arab countries to identify factors contributing to research success. The Arab League includes countries that differ significantly in the Gross Domestic Product (GDP) and Human Development Index (HDI). The Gulf Cooperation Council (GCC) nations are categorized as high-income countries. They have an average GDP of $15,000. In contrast, Arab countries like Somalia and Sudan have a GDP of $250. While some Gulf nations have high economic indicators, they have a shortage of comprehensive development factors like health and education (12, 13).

Our study revealed that publications output for the 25 years covering 1997–2022 from the Arab countries revealed an average annual growth rate of 11.5% with an average output of 5.7 articles per million population. However, on average, the Arab countries had a research productivity per capita that was 24-fold less than developed countries from Europe, Canada, and the United States. In addition, our study revealed that Arab countries exhibited research productivity per medical school that was 15 times lower than that of the United States and Europe. There are certain underexplored avenues for research in the Arab world. For example, consanguinity rates are notably high, and there is an opportunity to explore research related to the genetic basis of congenital heart disease. Studies have emphasized that consanguinity poses a significant risk factor and have advocated for more genetic research in this domain (14, 15). Although a few research centers in the Arab world have been active in researching the genetics of congenital heart disease, there is a critical gap in research concerning this topic, necessitating more focused investigations.

There was a significant variation in the research output between the various Arab countries. Five Arab countries produced 78% of the publication output. The high-income countries (HICs) had higher productivity than the low and middle-income countries (LMICs). The lower-middle-income countries in North Africa had in-between productivity between LIC and HIC. The post or in-conflict countries like Iraq and Libya had low productivity, although they rank as upper-middle-income countries. The research productivity in non-western and non-Arab countries in Africa and Asia exhibited a similar pattern to that observed in Arab countries. It correlated with a country's GDP, as evidenced by the research productivity in Pakistan, India, Nigeria, and South Africa. While higher GDPs correlate with increased research productivity, it's essential to recognize that various other factors, including human resources, government vision, systematic development, country stability, and fostering a research culture, significantly contribute to the promotion of research.

The most cited Arab countries were Lebanon, Egypt, and Saudi Arabia, with an average citation per article ranging between 10.1 and 7.5, which was 15 times higher than that reported from the LICs in the Arab world. Most published articles were clustered in Quartile 3 (*Q*3) journals, with very few published in Quartile 1 and *Q*2. Among the ten most common journals in which the articles were published, half were journals published in the region. Latif reported an increasing trend of publishing medical and biomedical research in local journals with a low impact factor in the Arab world (16).

About one-quarter of the published articles (26%) involved multiple countries' production and international co-authorship. The increase in productivity output was associated with an increase in multiple countries' cooperation with the US and Europe and an increase in international authorship. This was also associated with an increase in the published article's impact, as reflected by an increase in citations. Research in developing countries can have a higher impact through a more significant proportion of citations when there is international collaboration with developed countries. Of noteworthy significance is the role of diaspora collaborations, as reflected by the list of involved authors, as a key determinant of success.

Among the most relevant and frequent affiliative and universities involved in the publications, twelve were from the region, four from France, two from the USA, and one each from Canada and the UK. The study identified pockets of good-caliber research programs with relatively good productivity, representing universities from Egypt, Saudi Arabia, and Lebanon, with the universities from the high-income countries having articles with higher impact and citations.

The published research by the Arab countries had been predominantly clinical research. The most common topic was the management of children with congenital heart disease. Heart disease's genetic and molecular basis were the least addressed topics. Innovative, basic, and applied research in pediatric cardiology in the Arab world faces obstacles and limitations. Distribution of publications by country or economy can indicate research priorities and capabilities. Farhat et al. noted that research output on (CHD) per capita in the Arab world was notably low, estimated to be 29 times less than that of developed countries in 2013 (17). Over the past decade, there has been progress in publication output per capita. Notably, the absence of significant research focusing on adults with CHD in our study signifies a substantial gap in our understanding of the progression and management of CHD into adulthood. Despite the growth, research impact, quality, and innovation have major challenges.

Improved research productivity and impact in Pediatric and Congenital Heart Disease in select Arab nations reflect government support and vision. For instance, Gulf Cooperation Council countries have invested in infrastructure, universities, and human resources, enhancing research output (18). Similarly, in Egypt, government initiatives, such as the Egyptian Knowledge Bank (EKB) (19), have promoted research, as evidenced by the number of articles produced by this institution (Figure 7). In some middle-income countries, like Lebanon, private non-profit universities have significantly contributed to research advancement, as seen in their high citations and productivity. These successful programs are built on government and university commitment, resource allocation, facility provision, and fostering growth (17, 20).

Private institutions and philanthropic partnerships are also instrumental in advancing pediatric cardiac surgery programs in resource-limited countries. This collaborative approach, involving public, private, and philanthropic entities, has led to establishing high-quality programs with outcomes comparable to those in developed countries, as evident in the case of Lebanon (20). Such models highlight the value of diverse funding sources and institutional support in enhancing research and clinical care in the field of CHD. The call for multinational studies in the Arab world to develop good pediatric cardiac services highlights the importance of collaborative efforts supported by governments and universities in the region (21). Most funding for research on congenital heart disease in the Arab world comes from the USA and Europe, with minimal funding from the region.

To enhance research infrastructure and output on pediatric and congenital heart disease (CHD) in the Arab world, addressing the issue of access to care is critical. Global healthcare access disparities significantly impact the prevalence and management of CHD in these regions, particularly in light of the unique socio-economic, cultural, and political dynamics (3). Addressing the issue of access to care in the Arab world should consider the specific challenges and opportunities within the region. This may involve conducting comprehensive needs assessments to identify gaps in healthcare infrastructure, evaluating the capacity of existing healthcare systems, and exploring potential barriers to accessing specialized care for pediatric patients with CHD (22).

Creating robust Pediatric and Congenital Heart Disease research in the Arab world is feasible. Challenges must be addressed, and collaborations play a key role. Every institution and country should evaluate its resources and formulate an approach that best aligns with its needs and challenges. The results of this study shall provide data for policymakers, educational institutions, and governments, equipping them to discern the components integral to cultivating a research program and fostering a research culture while simultaneously tackling associated challenges.

While our study offers valuable insights, it is not without limitations. Relying on data from selected databases and the methodologies of the Bibliometrix Package may introduce biases, primarily focusing on quantitative aspects. Future research should explore qualitative intricacies and broader impacts. The dataset encompasses Arab and non-Arab countries, recognizing collaborative efforts but introducing complexities in regional interpretation. It is crucial to note that while international collaborations are essential for advancing regional research, strategic decisions should guide these partnerships to enhance research and higher education system capacity rather than creating virtual affiliations. Recent reports revealing a decline in top researchers affiliated with Saudi Arabian universities following attempts to boost rankings underscore this concern (23). Despite these challenges, our study, guided by comprehensive science mapping analysis and inclusive criteria, provides a comprehensive overview of the congenital heart disease research landscape. We recognize the necessity for further qualitative exploration and consideration of collaboration dynamics.

## 5 Conclusion

Significant disparities exist in pediatric and congenital heart disease research on regional and global scales, particularly between developing and developed nations. Research output varies significantly by country, with diverse impacts and citation rates. Arab countries' research productivity and impact appear closely tied to GDP per capita, while conflict-affected nations demonstrate lower productivity despite acceptable GDP levels.

Enhancements in pediatric cardiology research productivity and impact within specific Arab countries result from factors such as robust government support, systematic development, country stability, ample funding, international collaborations, and a well-defined research vision. We encourage each country to identify its strengths and tailor its research system, considering available resources.

## Data Availability

The original contributions presented in the study are included in the article/Supplementary Material, further inquiries can be directed to the corresponding authors.
